# MicroRNA-485-5p targets keratin17 to regulate pancreatic cancer cell proliferation and invasion via the FAK / SRC / ERK pathway

**DOI:** 10.7150/jca.90689

**Published:** 2024-02-17

**Authors:** Peng Chen, Meng Pan, Zhengchao Shen, Yuquan Yang, Xiaoming Wang

**Affiliations:** 1 Department of Hepatobiliary Surgery, The Second Affiliated Hospital of Wannan Medical College, Wuhu, Anhui 241000, P.R. China.; 2 Department of Hepatobiliary Surgery, The First Affiliated Hospital of Wannan Medical College, Wuhu, Anhui 241001, P.R. China.

**Keywords:** Pancreatic cancer, KRT17, MiRNA-485-5p, Cell cycle, Proliferation

## Abstract

**Background:** It is crucial to probe into the biological effect and mechanism of miRNA-485-5p regulating keratin 17 (KRT17) in pancreatic cancer (PC) to understand its pathogenesis and identify potential biological targets.

**Methods:** The bioinformatics means were used to evaluate the clinical significance of KRT17 expression in the Cancer Genome Atlas (TCGA) database. TargetScan database analysis in conjunction with dual luciferase and RNA Immunoprecipitation (RIP) experiments was used to probe the interaction relationship of miRNA-485-5p with KRT17. The expression of miRNA-485-5p and KRT17 in PC tissue and cancer cell lines was detected by Q-PCR paired with western blot assay. The biological function of miRNA-485-5p in regulating KRT17 was investigated in the PC cell line via gene silencing/overexpression technique. A western blot experiment was utilized to investigate the regulatory effect of KRT17 on cell cycle-related proteins and the FAK/Src/ERK signal pathway.

**Results:** The level of KRT17 was increased in PC tissues and this significantly decreased the survival rate of PC patients. TargetScan in combination with dual luciferase and RIP experiments verified the miRNA-485-5p target KRT17. The expression of KRT17 was high in the PC cell line, although the expression of miRNA-485-5p was low. Silencing KRT17 or overexpression of miRNA-485-5p significantly inhibited PC cell viability, proliferation, invasion, and colony formation, while promoting apoptosis. Overexpression of KRT17 drastically reversed the function of miRNA-485-5p. The silenced KRT17 remarkably downregulated the expression of cyclinD1, Cyclin Dependent Kinase 1 (CDK1), CDK2, Phospho-Focal Adhesion Kinase (p-FAK), p-Src, and p-ERK proteins in the PC cells.

**Conclusion:** Generally, an essential signaling cascade of miRNA-485-5p/KRT17/FAK/Src/ERK influences the biological functions of PC cells.

## Introduction

Pancreatic cancer (PC) severely impacts the lives of patients 1. The prognosis is mainly determined by the comprehensive evaluation of clinical and pathology during diagnosis 2. Although clinical therapies and surgical resection can predict PC patient survival, patients with the same clinicopathological features may have different clinical prognoses 3. This implies that other uncertain characteristics of PC may lead to these disparities. Currently, clinically, surgical resection of tumor tissue, chemo - and radiotherapy treatments for patients, and so on, have been the primary modality of choice for the treatment of PC 4-6. Unfortunately, the curative effect is bad, and the 5-year survival rate is quite low. It is mostly due to having no adequate knowledge of the pathogenesis of PC, therefore complete research of the mechanism of PC, particularly the discovery of new core molecules and their mechanisms, will help to improve the prognosis of PC 7-9.

Since its discovery, keratin (KRT), an essential factor in normal angiogenesis, and many genes in the keratin family have been indicated to act a vital regulatory role in tumor cells 10. Keratin is further divided into “type I and type II” based on the protein sequence encoded by keratin 11-13. Keratin 17 (KRT17) in type I keratin is related to the process of happening and advancement of many kinds of sorts of tumors 14. KRT17, for example, not only stimulates the growth of skin tumors but is also highly expressed and enriched in oral squamous cell carcinoma 15, 16. Proteomic studies and subsequent verification revealed the overexpression of the KRT17 protein in malignant tumors, which is consistent with the results of RNA screening 17. Accumulated evidence suggests that KRT17 is also abnormally expressed in cervical squamous cell carcinoma and gastric cancer. The mechanism of KRT17 regulating tumor progression is tissue specific 18, 19. For instance, KRT17 enhances the proliferation of oral cancer by up-regulating Akt/mTOR signal pathway and suppresses the content of cleaved-caspase-3 to strengthen its function 20. Tissue-specific cytokine polarization can be induced by low expression of KRT17 in cervical cancer and it is necessary to recruit effector immune cells to vulnerable cervical epithelial cells to prevent the growth of cancer 21. Despite some studies that have discovered an increase in the expression of KRT17 in PC, the function and mechanism of KRT17 in this disease still need to be clarified.

Although the pathogenesis of PC is not completely understood, much research has discovered that microRNA (miRNA) is abnormally expressed in PC 22. Moreover, it participates in the biological regulation of PC, and act an essential role in carcinogenic or tumor suppressor genes, which is expected to become a new biomarker 23. MiRNAs repress target genes by participating in the preventing translation process of target mRNAs 24. According to some studies, miR-485-5p is downregulated in many types of malignancies, and altering miR-485-5p expression can have some influence on the process of tumor invasion and metastasis 25. For example, miR-485-5p was expressed at very low levels in both osteosarcoma, colon cancer, and other malignant tumor cells, and act a vital role in tumor cell proliferation, invasion, migration, and other functions 26, 27. According to the report, up-regulated its expression can make hepatocellular carcinoma cells less able to proliferate, invade and migrate 28. MiR-485-5p has the potential for diagnosis and treatment in tumors based on a variety of indicators.

The objective of this research is to detect the expression of miR-485-5p and KRT17 in PC samples and cell lines and to analyze the interaction of miR-485-5p and KRT17 in PC cell lines and its potential mechanism to provide experimental support for targeting miR-485-5p/KRT17 axis in the treatment of PC.

## Materials and methods

### Clinical samples

In the present research, 20 cases of PC and adjacent non-tumor specimens (10 females and 10 males) were collected after surgical resection in the First Affiliated Hospital of Wannan Medical College from November 2020 to December 2022. All patients had no history of radiotherapy, chemotherapy, targeted therapy, or other pancreatic diseases before the operation. The pathological samples of patients were clinically assessed by two experienced pathologists.

### Cell culture

The human PC cell lines PANC-1 and SW1990, the human immortalized pancreatic duct epithelial cell line H6c7 cells were obtained from Guangzhou Cellcook Cell Biotechnology, Ltd (http://www.cellcook.com). All cells in 100 μg/mL streptomycin, 100 U/mL penicillin, and 10% fetal bovine serum (Gibco, Carlsbad, CA, USA) were cultured in RPMI-1640 medium (Hyclone Laboratories, South Logan, UT, USA) and cultured in an incubator (5% CO_2_, 37 °C).

### The siRNA and plasmid transfections

Plasmid and siRNA were transfected into cells by Lipofectamine™ 3000 (Thermo Fisher Scientific) for 48~72 hours. The transfection-related reagents were prepared and marked as A and B tubes. For A tubes, each well was 5 μL Lipo3000 transfection reagent plus 125 μL RPMI-1640 medium mixed. For B tubes, in each well, 125 μL RPMI-1640 medium, 1-2.5 μg DNA, and 2-5 μL P3000 (transfection of siRNA was without P3000) were mixed. The liquid in the A tube was mixed with the liquid in the B tube and slowly added to wells in 6-hole plates. After addition, the contents were mixed by shaking the plates back and forth slightly, followed by incubation at a constant temperature for 48~72 hours.

### Cell viability assay and observation of cell morphology

The cells (1.8 × 10^3^/well) were inoculated into 96-well plates (three plates). After 24 hours of culture, the degree of cell confluency was about 50%, then 10 μL of CCK8 (Beyotime Institute of Biotechnology, Suzhou, China) and 90 µL RPMI-1640 complete medium were added. After being cultured for 2~4 hours in an incubator, the absorbance of the cells was tested using an enzyme labeling detection instrument at 450 nm (BioTek, Winooski, VT, USA).

### Dual-luciferase reporter gene assay

The binding site sequences (Targetscan database) were amplified and separately constructed into the reporter vector. Using Lipofectamine™ 3000 transfected vector into chondrocytes. Following the procedure described in the dual luciferase kit instructions (Guangzhou Ruibo Co., Ltd., Guangzhou, China). Ie., cells were collected 48 h after transfection, lysed by adding cell lysis buffer, and the supernatant was collected after centrifugation and added 50 uL luciferase assay reagent, shaken and mixed for 10 min to measure dual luciferase activity, respectively, in term of the fluorescenogsintensity, the comparative luciferase activity of cells in each tissue was calculated (Promega, Madison, WI, USA).

### Flow cytometry detection

Cells were collected after 48 hours of transfection in a six-well plate (the cell density was about 5 × 10^5^). The buffer solution was prepared according to Binding Buffer (10 ×) 10 μL + D_2_H_2_O 90 μL per well. Transfer the supernatant of the 6-orifice plate into a 15mL centrifuge tube. Then clean the 6-hole plate with PBS twice. In each hole is added 0.25% EDTA-free trypsin (preheated) 1mL digestion, centrifuged 5min. A binding Buffer of 100 μL is added to each centrifuge tube, mix well and avoid light for 15min. Add 250 μL of Binding Buffer and mix well. Each tube is added Annexin V-FITC/PI 1 μL (BestBio, Shanghai, China). The apoptotic levels of cells were informed by the flow cytometry results.

### Detection of cell clone formation by Giemsa method

The treated cells of each group were seeded in the plate (400-1000/well), and cultured in an incubator (5% CO_2_) for 14 days. Fixed the cells with paraformaldehyde fixation solution (Beyotime), and then stained for 10 min with a rapid Giemsa staining kit (Thermo Fisher Scientific). Finally, using a microscope and photographed to count the number of cell clones(> 50 cells/clones).

### Transwell migration test

Matrigel was coated into the apical cavity of a Transwell chamber for invasion assay (Thermo Fisher Scientific). Cells (2 × 10^5^/mL) were cultured for 12 to 24 hours in the absence of serum. 600 uL of the medium was added to the lower chamber (10% FBS), and the cell suspension was seeded in the upper chamber. Rinsing was performed after 48 hours, and 800 uL of methanol was mixed in 24 well plates, to make sure the upper chamber solution has been blotted dry (4% paraformaldehyde). 800 uL of staining solution was mixed with another plate. After the staining solution was completely washed and the fixative solution on the upper chamber was blotted, it was transferred into 0.1% crystal violet staining solution, and then taken to be photographed under an inverted microscope to observe the changes of cell invasion ability in each group (20 min staining at room temperature).

### 5-Ethynyl-2'-deoxyuridine (EdU) test

Each group of cells to is grown to approximately 80% cell density in a 96-well plate. An equal volume of EdU solution (Thermo Fisher Scientific) was added to 500 mL medium per well and allowed to act for 2 h. Fixative was added for 0.5 h after discarding the culture medium. This was followed by rinsing (3 times / 5 min) with PBS. The permeabilization solution (100 uL/well) was added and cultured for 15-20 min. This was followed by rinsing (2 times / 5 min) with PBS. Cells were then cultured for 30 min (Apollo staining solution and DAPI). Fluorescence microscopy was performed with a microscope in five randomly selected areas to analyze the proliferation rate (Nikon Corporation, Japan).

### Q-PCR

Extraction of RNA from tissues involved grinding the tissues in a grinding disruptor, then approximately 1 mL TRIzol lysis solution (Tiangen Biotech, Beijing, China) was added for every 50 mg of the tissue sample. To extract the RNA from cells, added 1 mL TRIzol lysis solution to each well. A total of 800 µL of 75% ethanol was added. Diethylpyrocarbonate (DEPC) water was then added, followed by mixing with gentle pipetting. The extracted RNA was later reverse-transcribed into complementary DNA (cDNA). Each group of cDNAs and amplified primers (Ribobio, Guangzhou, China) were co-incubated with SYBR fluorescent dye (Tiangen Biotech). The mixed solution was later added to an 8-tube strip and put in a fluorescence quantitative PCR instrument for PCR amplification, and the relative abundances of RNAs were computed using the 2^-ΔΔCt^ method (Table 1).

### Western blotting

According to the extracted protein molecular weight size by Protein Extraction and Quantification Kit (Thermo Fisher Scientific), different concentrations of separating glue were selected, after TEMED (Merck & Co., Inc.) was added, the mixture was injected into the glue maker, and the glue was sealed using methanol; After the separating gum has set, slowly pour off methanol from one side of the glass plate and choose an appropriate volume of concentrating gum. After TEMED was added and similarly injected into the glass plate, subsequently the sample comb was slowly and vertically inserted into the concentrated gum solution and left for 20-30 min; After the concentrated gel solidified, the sample comb was drawn out, and the configured electrophoresis liquid was added into the inner and outer troughs of the glass plate, forming a closed loop; Add protein maker and protein sample, 20 UG / well, to the comb tank with a pipette. Note the inner tank electrophoresis liquid needs to be filled, the outer tank is over 5 cm from the bottom; After loading, the connected electrophoresis instrument power supply, set the instrument parameters, the concentrator was 80 V, and the separating gum was 120 v. After electrophoresis with loading buffer to the bottom of the gel, stop electrophoresis, turn off the power supply: cut a PVDF membrane of suitable size according to the gel size and immerse it with methanol for 10 min; Gently disassemble the glass plates and remove the gel; Multi empty pads, thick filter paper, gel (black plate), PVDF membrane (white version) were placed on the splint in sequence according to the "black glue white membrane", and the splint constructed from the "sandwich structure" was inserted into the transmembrane tank at a constant current of 240 MMA for 120 min; The PVDF membrane was removed from the splint and placed into an antibody incubation tank containing Western blocking buffer; Tbst buffer wash 5 times, 5 min / time, the antibodies against KRT17, cyclinD1, CDK1, CDK2, phosphorylated (p)-FAK, FAK, phosphorylated (p)-Src, Src, phosphorylated (p)-ERK, ERK, and β-actin (Cell Signaling technology, Inc.) were poured into the antibody incubation tank, respectively, and the refrigerator at 4 ℃ overnight: tbst buffer wash 5 times, 5 min / time; Diluted secondary antibody (1:3000; Abcam, Cambridge, Ma, USA) working solution was added into the incubation tank for 3-4h at room temperature shaker; Wash 4 times with tbst buffer, 5 min / time; Luminescent detection solution was configured in a 1:1 ratio; Forceps after removing the membrane and blotting the excess liquid on the membrane, luminescence detection solution mixed with PVDF membrane, and placed it into a phosphorimager and imaged (BioRad).

### Statistical analysis

All data were analyzed using SPSS statistical software for Windows, version 18.0 (SPSS, Chicago, IL, USA). The results showed a normal distribution with homogenous variance, expressed as the mean ± standard deviation. We used a single sampling *t*-test to compare data from the two populations and a one-way analysis of variance. *P* < 0.05 was considered a statistically significant difference.

## Results

### Expression of KRT17 in Pancreatic Cancer (PC) and normal Pancreatic tissues in TCGA Database

**Figure 1:**
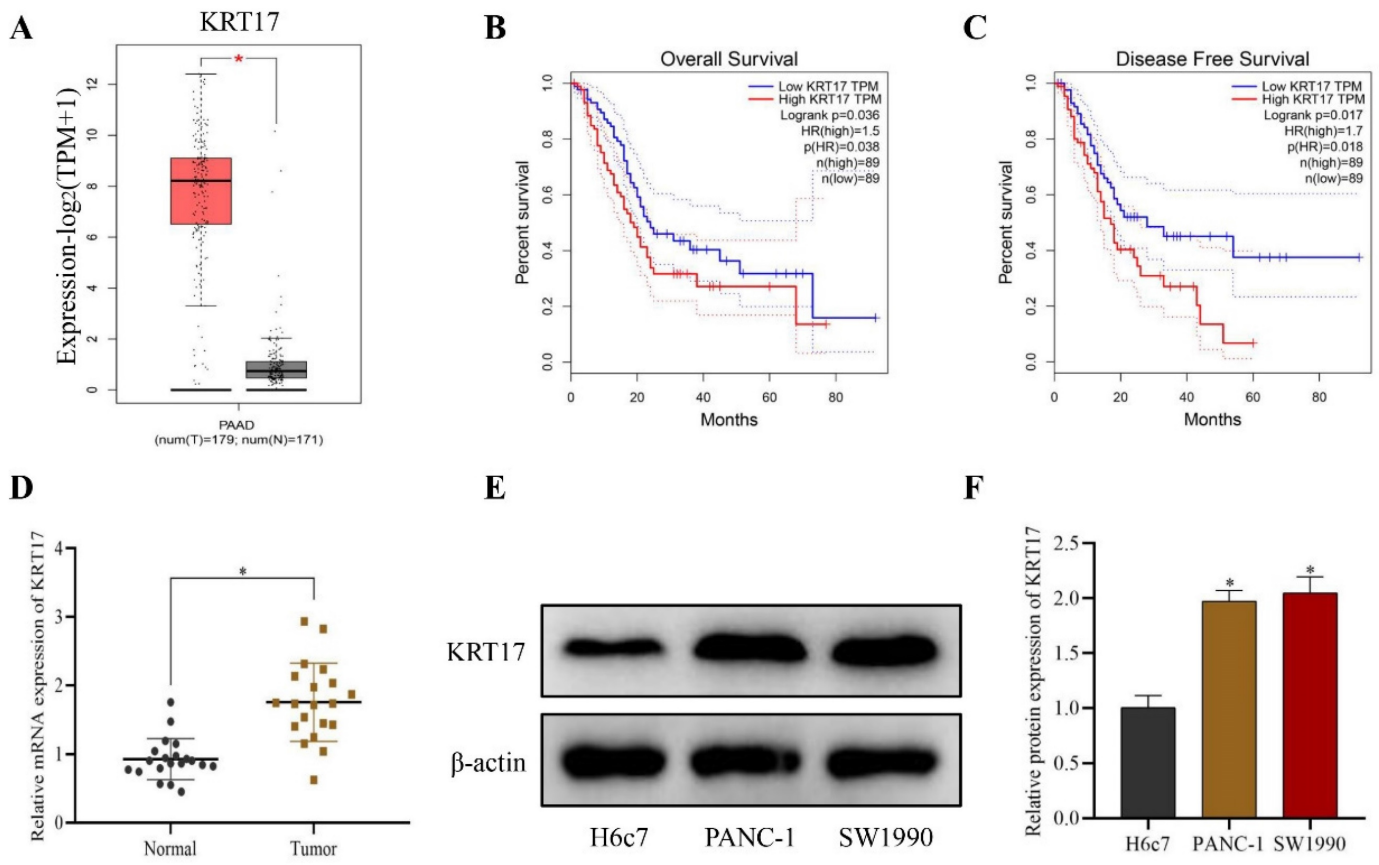
Expression level and Clinical significance of KRT17 in Pancreatic Cancer. **A** The expression of KRT17 in the TCGA database. **B** Analyses of the Overall Survival of KRT17 expression. **C** Analyses of the Disease-Free Survival of KRT17 expression. **D** Detection of KRT17 expression in Pancreatic Cancer and healthy human pancreas by Q-PCR. **E** Immunoblots of KRT17 and β-actin in cells. **F** The statistical plot of KRT17 protein expression. ^*^P < 0.05 vs. Normal or H6c7.

TCGA database indicated that the level of KRT17 in pancreatic cancer was increased than in normal pancreatic tissues (*P* < 0.05) (Figure 1A). After analyzing the association between survival data and KRT17 expression through the TCGA database, it was found that the overall survival rate (*P* = 0.038) and disease-free survival rate (P = 0.018) of PC patients decreased significantly when KRT17 expression was high (*P*<0.05) (Figure 1B-C). Q-PCR experiment demonstrated that the level of KRT17 in PC tissue (n = 20) was substantially higher than in normal pancreatic tissue (n = 20) (*P* < 0.05) (Figure 1D). Western blot assay found that the content of KRT17 in PANC-1 and SW1990 cell lines was higher than that in H6c7 cells (*P* < 0.05) (Figure 1E-F).

### Effect of KRT17-siRNAs transfection on KRT17 silencing efficiency and cell viability in pancreatic cancer (PC) cell line

**Figure 2:**
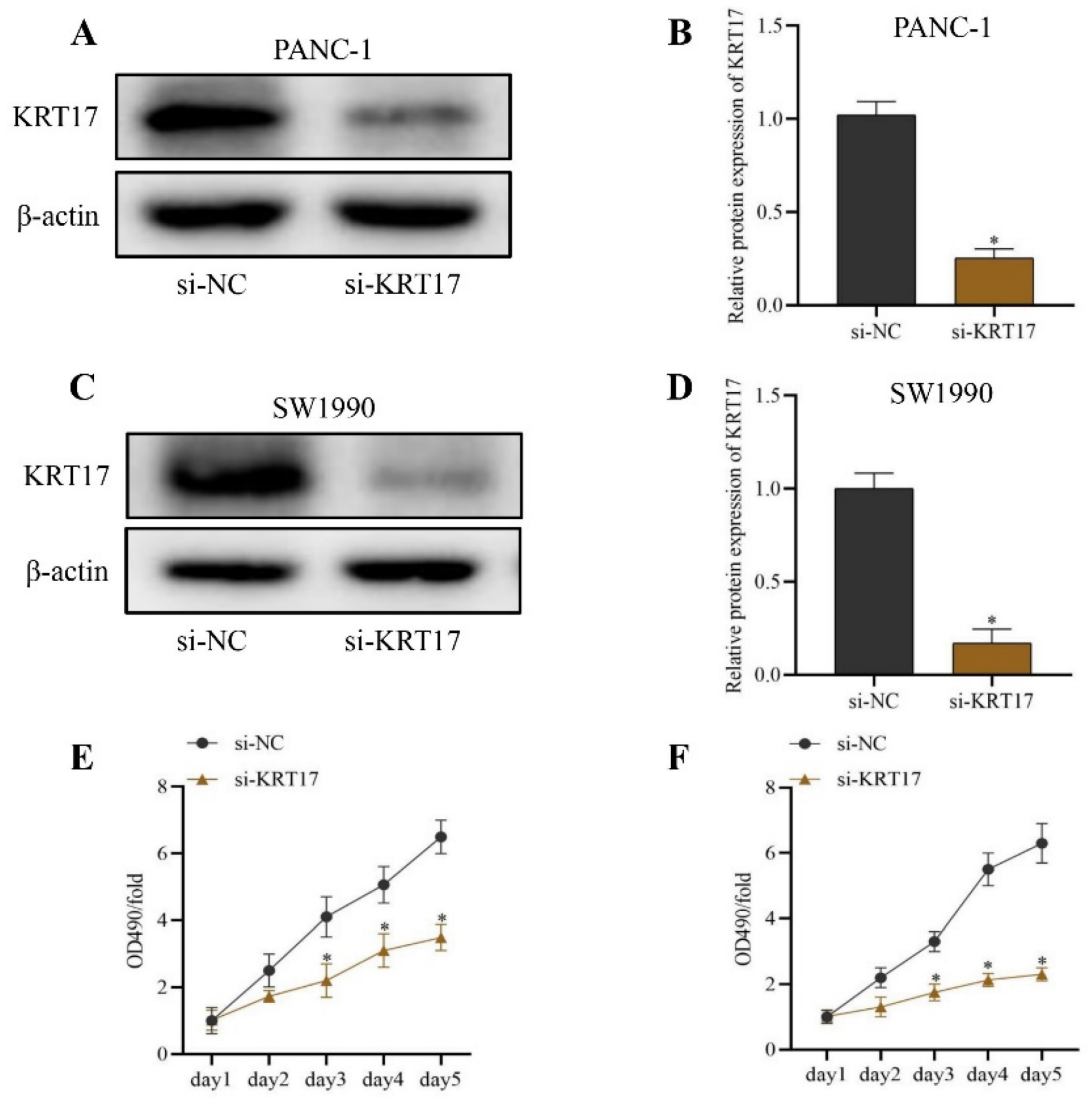
Silencing efficiency of KRT17-siRNAs in Pancreatic Cancer (PC) Cell Line and its effect on cell viability. **A** The immunoblots of KRT17 and β-actin in PANC-1 cell. **B** The statistical plot of KRT17 protein content. **C** The immunoblots of KRT17 and β-actin in SW1990 cell. **D** The statistical plot of KRT17 protein content. **E** The influence of silencing KRT17 on PANC-1 cell viability. **F** The influence of silencing KRT17 on SW1990 cell viability. ^*^P < 0.05 vs. si-NC.

The outcome of western blot indicated that the content of KRT17 protein in the si-KRT17 group was lower than that in the si-NC group to detect the transfection efficiency of KRT17-siRNA in PC cell lines (*P* < 0.05) (Figure 2A-D). Similarly, the outcome of the CCK-8 test indicated that the cell viability of the si-KRT17 group was significantly lower than that of the si-NC group in the PC cell line (*P* < 0.05) (Figure 2E-F).

### Effect of KRT17-siRNAs transfection on apoptosis and invasion of Pancreatic Cancer (PC) Cell Line

**Figure 3:**
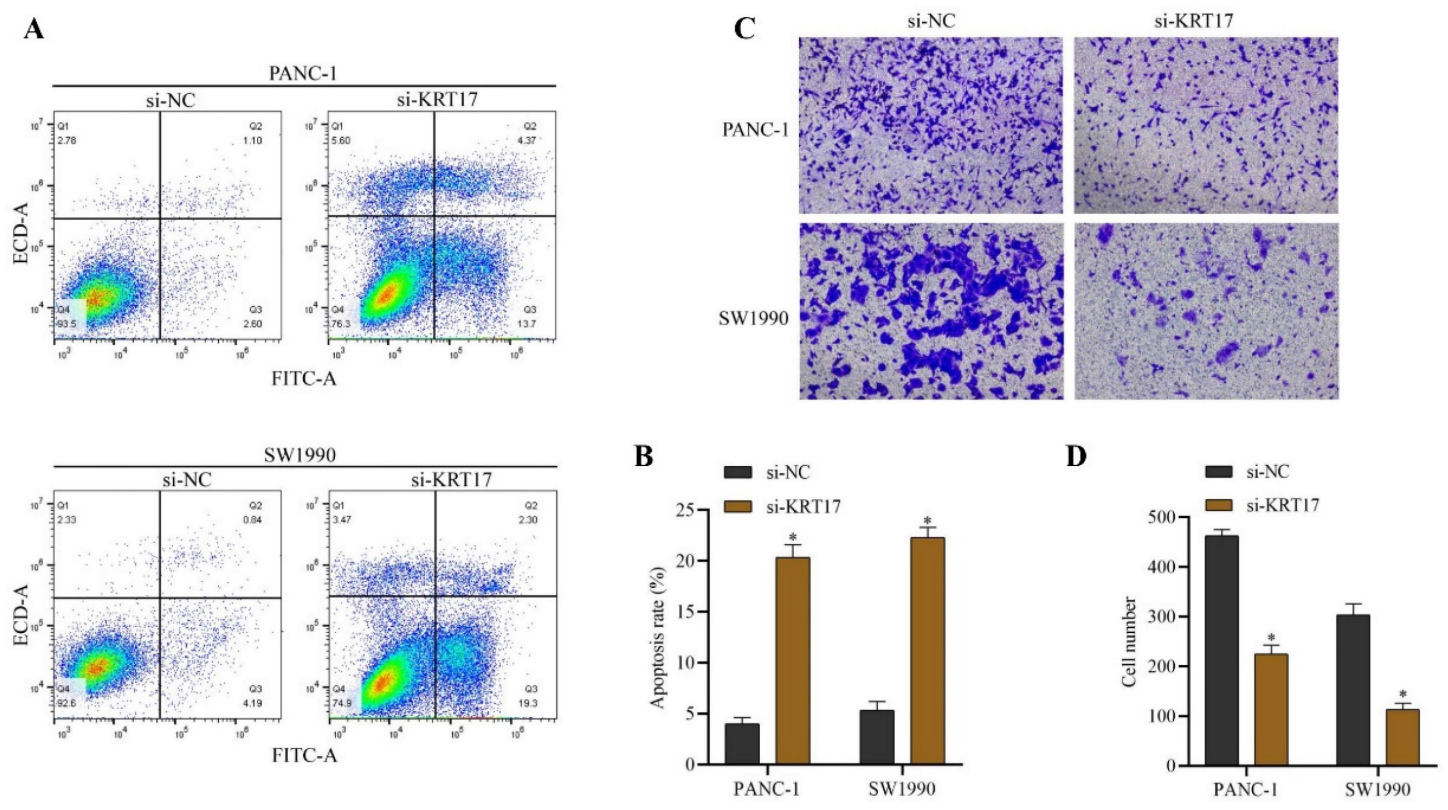
Effects of inhibition of KRT17 gene expression on invasion and apoptosis of cells. **A** The influence of silencing KRT17 on apoptosis rates of PC cells detected by flow cytometry experiment. **B** Statistical chart of apoptosis rate. **C** The effect of silencing KRT17 gene expression on the invasion of PC cells was observed by the Transwell experiment. **D** The statistical plot of the invasion number of cells. ^*^P < 0.05 vs. si-NC.

Flow cytometry analysis indicated that the incidence of apoptosis of PC cells increased after si-KRT17 treatment (*P* < 0.05) (Figure 3A-B). The experiment of Transwell revealed that silencing KRT17 in PC cells markedly inhibited the invasive capacity of the cells (P < 0.05) (Figure 3C-D).

### Effects of KRT17-siRNAs transfection on cell proliferation and colony formation in pancreatic cancer (PC) cell lines

**Figure 4:**
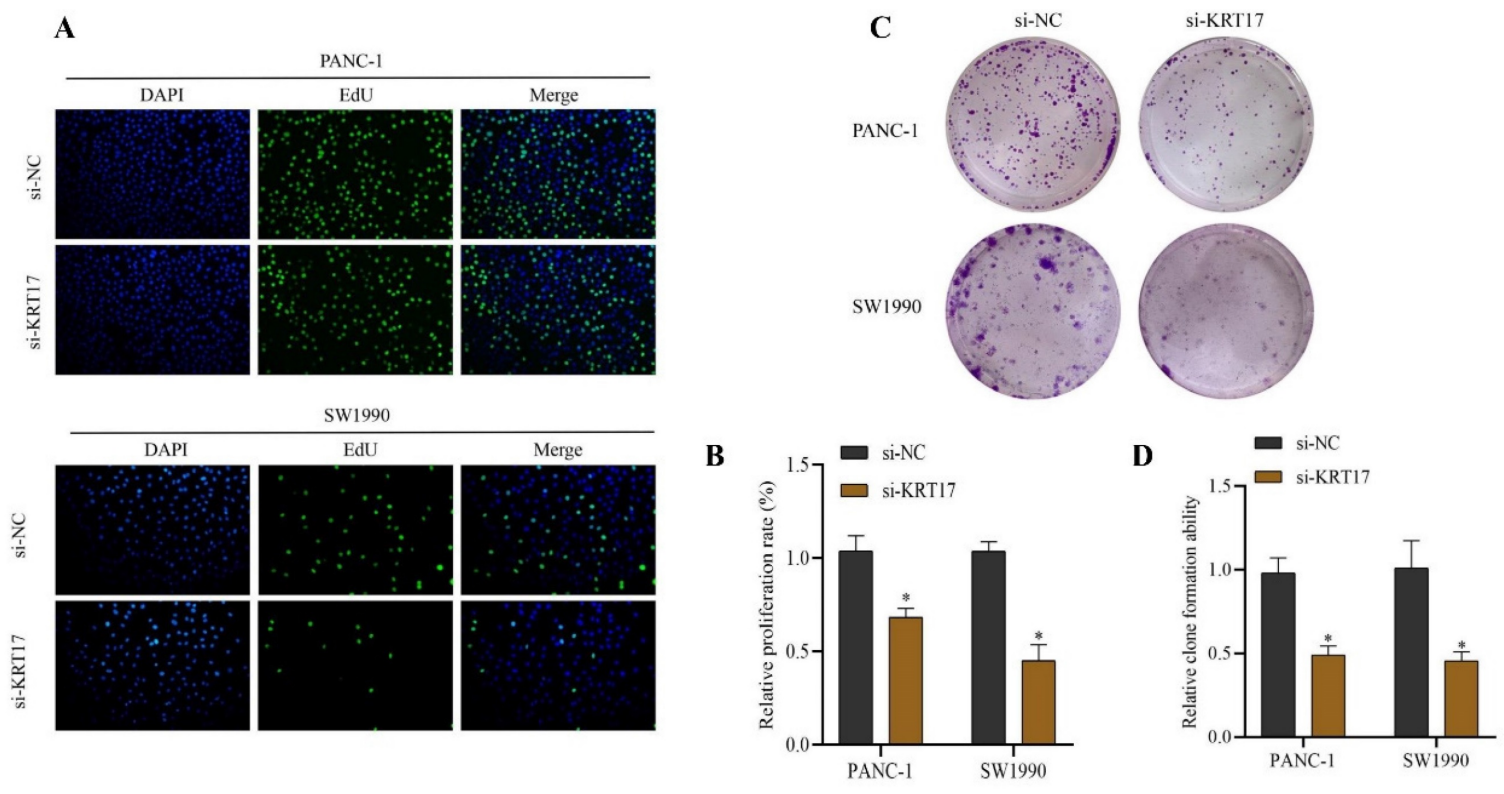
Influence of silencing KRT17 on the proliferation and colony formation ability of cells. **A** The EdU experiment was applied to detect the influence of silencing KRT17 on the proliferation ability in the cells. **B** The statistics plot of cell-associated proliferation rates.** C** The influence of silencing KRT17 on the clone-forming ability of cells was using the cloning experiment. **D** The statistical chart of the clone-formation ability of cells. ^*^P < 0.05 vs. si-NC.

The outcomes of the EdU experiment revealed that the proliferation rate of the cells after knockout KRT17 was lower than that in the si-NC group (*P* < 0.05) (Figure 4A-B). The clone-forming ability of the si-KRT17 group was lower than that of the si-NC group in cells cultured in vitro (*P* < 0.05) (Figure 4C-D).

### Effect of KRT17-siRNAs transfection on the expression of cell cycle-related proteins in pancreatic cancer (PC) cell lines

**Figure 5:**
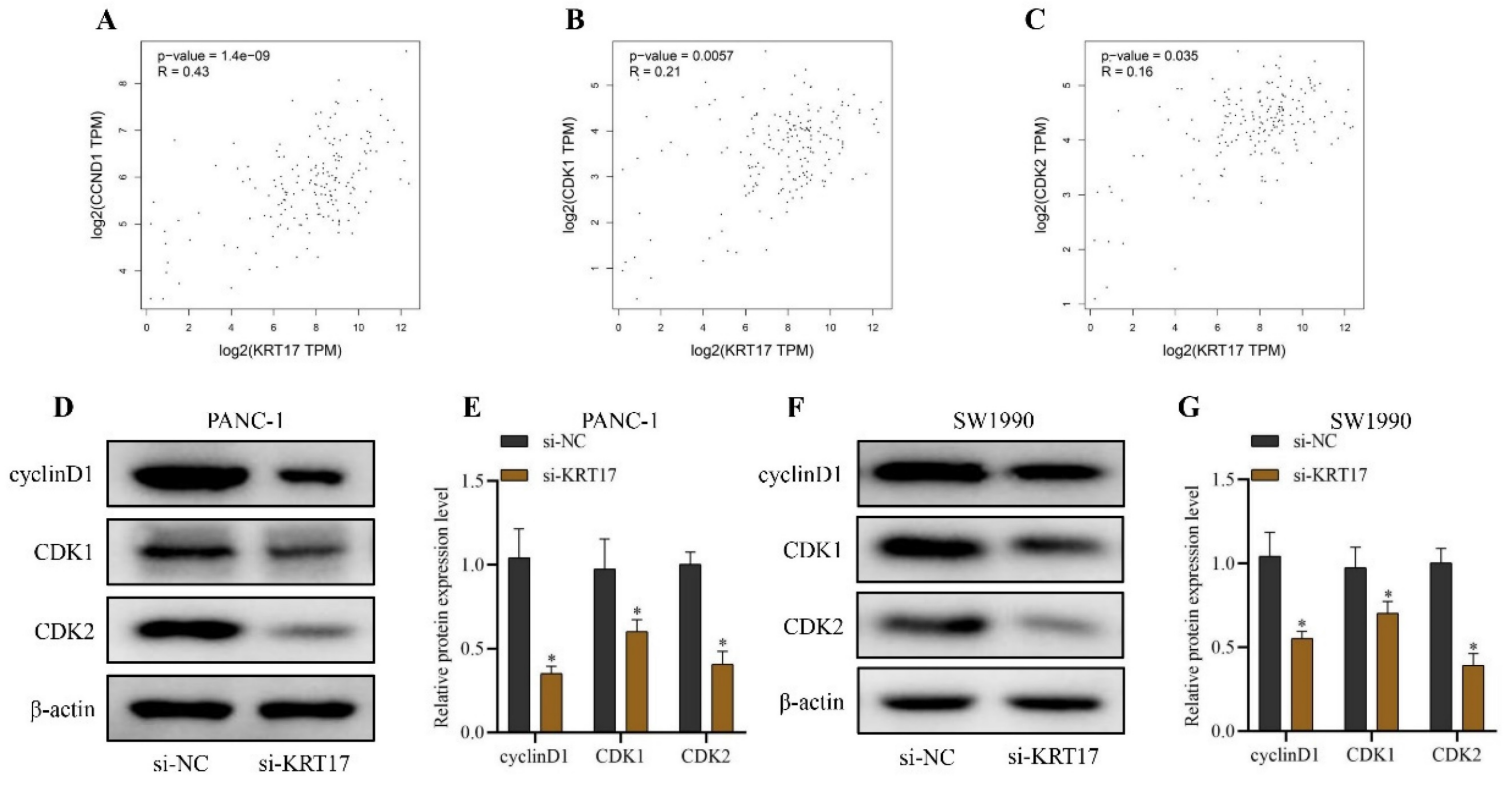
Analysis of the correlation between KRT17 and cell cycle regulatory molecules. **A** Scatter plots display the correlation between krt17 and CyclinD1 (CCND1) expression.** B** Scatter plots demonstrate the correlation between krt17 and CDK1 expression. **C** Scatter plots exhibit the correlation between krt17 and CDK2 expression. **D** The immunoblots of cyclinD1, CDK1, CDK2, and β-actin in PANC-1 by the western blot. **E** The statistical plot of cyclinD1, CDK1, and CDK2 protein expression levels in PANC-1; F The immunoblots of cyclinD1, CDK1, CDK2, and β-actin in SW1990 by the western blot. **G** The statistical plot of cyclinD1, CDK1, and CDK2 protein expression levels in SW1990. ^*^P < 0.05 vs. si-NC.

We initially examined the correlation between KRT17 and cyclinD1 (CCND1), CDK1, and CDK2 genes using the GEPIA database to verify the influence of KRT17 on cell cycle regulatory molecules in PC. KRT17 was revealed to be positively correlated with CyclinD1 (CCND1) gene (*P* < 0.05, R = 0.43), CDK1 gene (*P* < 0.05, R = 0.21), and CDK2 gene (*P* < 0.05, R = 0.16) in PC (Figure 5A-C). Likewise, silencing KRT17 in cells vastly down-regulated the content of cyclinD1, CDK1, and CDK2 proteins (*P* < 0.05) (Figure 5D-G).

### Effects of KRT17-siRNAs transfection in pancreatic cancer (PC) cell lines on FAK / SRC / ERK signaling

**Figure 6:**
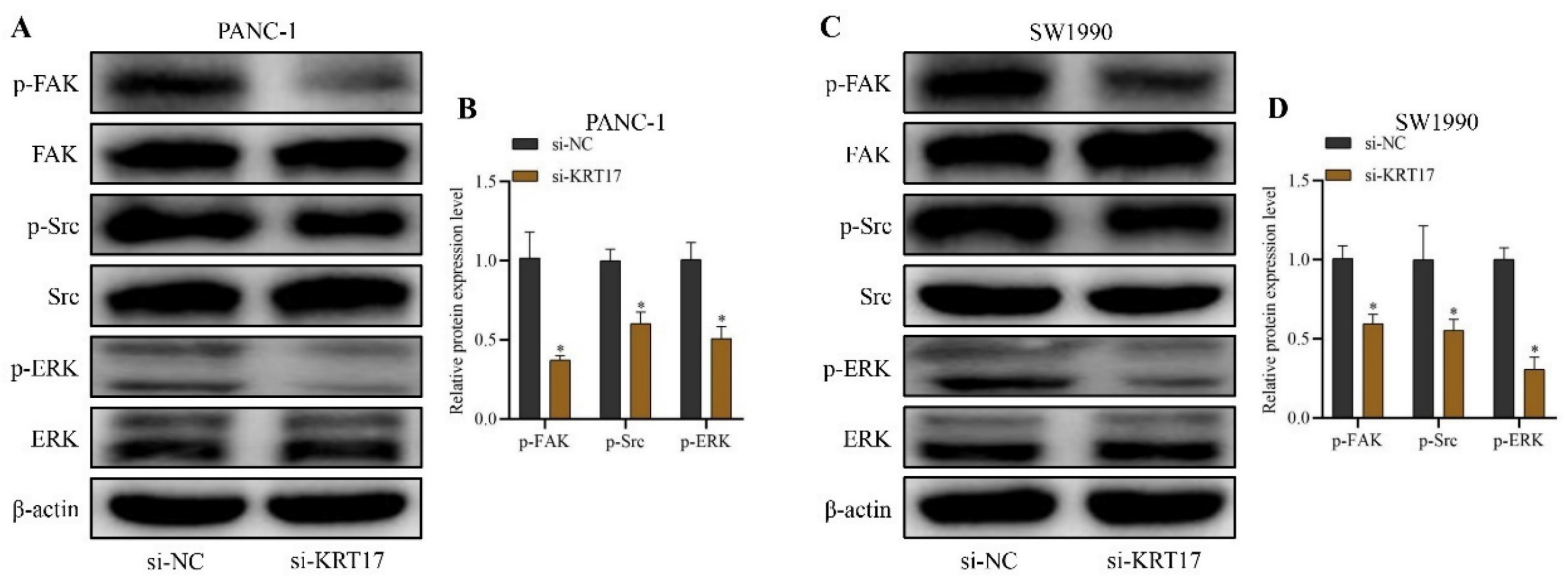
Effect of silencing KRT17 on FAK / SRC / ERK pathway. **A** Stand for the immunoblots of p-FAK, FAK, p-Src, Src, p-ERK, ERK, and β-actin in PANC-1 by the western blot. **B** The statistical plot of p-FAK, p-Src, and p-ERK protein expression levels in PANC-1. **C** The immunoblots of p-FAK, FAK, p-Src, Src, p-ERK, ERK, and β-actin in SW1990 by the western blot. **D** The statistical plot of p-FAK, p-Src, and p-ERK protein expression levels in SW1990.^ *^P < 0.05 vs. si-NC.

The influence of silencing KRT17 on the FAK/Src/ERK signal pathway was discovered by the western blot to explore the specific mechanism of the effect of KRT17 in PC. The outcome display that the protein content of p-FAK, p-Src, and p-ERK in cells was substantially lower than those in the si-NC group (*P* < 0.05) (Figure 6A-D).

### Interaction of miR-485-5p when targeting KRT17

**Figure 7:**
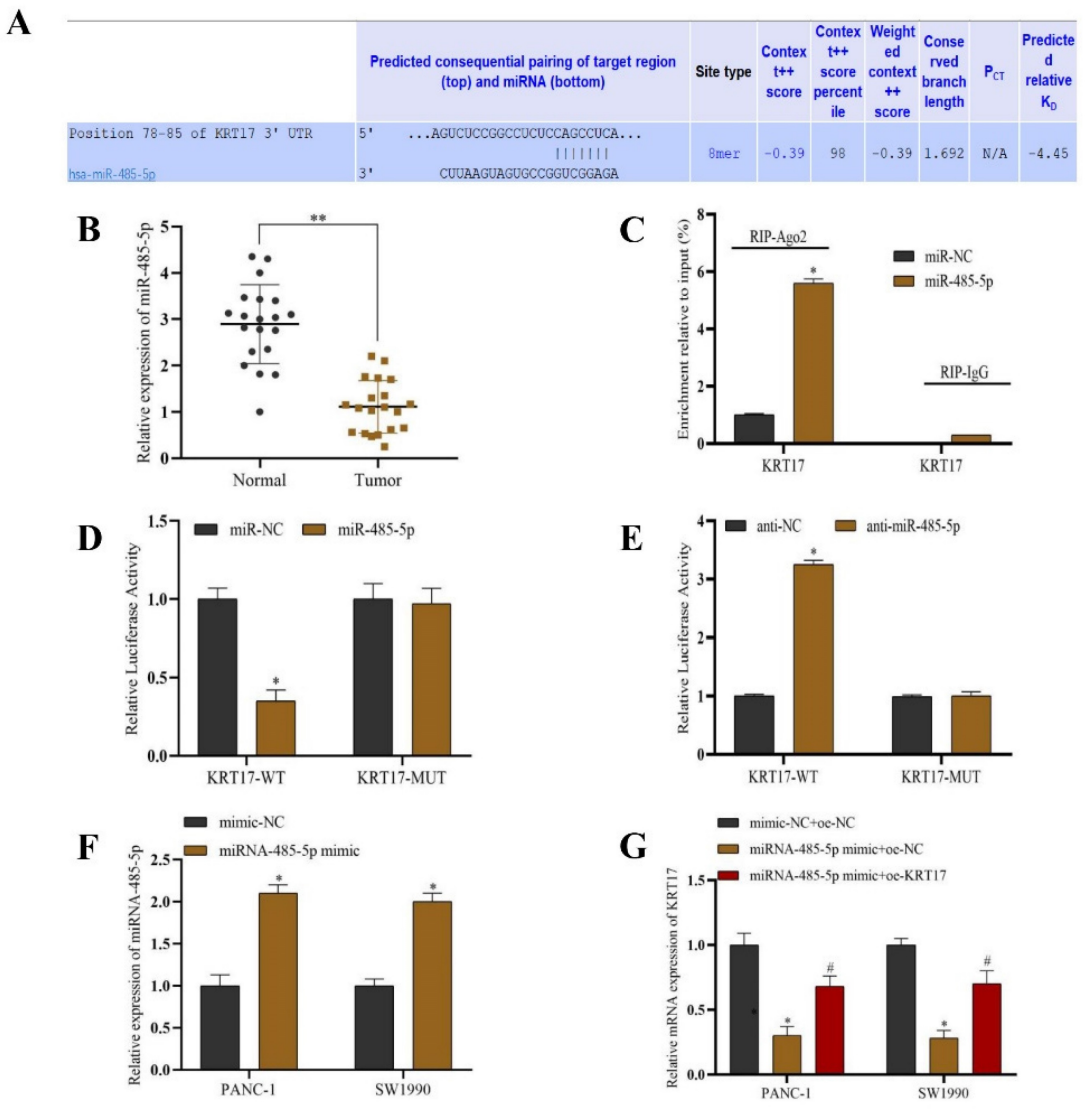
Validation of miR-485-5p association with KRT17. **A** The binding site of KRT17 was targeted by miR-485-5p in the TargetScan database. **B** The Q-PCR validation of miR-485-5p expression. **C** The correlation between miR-485-5p and KRT17 was detected by the anti-Ago2 RIP experiment. **D and E** A double luciferase reporter test proved the interaction between miR-485-5p and KRT17. **F** The transfection efficiency of miRNA-485-5p was determined by Q-PCR. **G** The influence of miRNA-485-5p on KRT17 levels was tested by Q-PCR. ^*^P < 0.05 vs. the Normal, miR-NC, anti-NC, or mimic-NC. ^#^*P* < 0.05 vs. miR-485-5p mimic+oe-NC.

We also utilized bioinformatics analysis to investigate the upstream miRNA targeting KRT17, which identified that KRT17 is an underlying target gene of miR-485-5p (Figure 7A). Q-PCR experiments revealed that the level of miR-485-5p in PC tissue (n=20) was considerably lower than that in normal pancreatic tissue (n=20) (P < 0.05) (Figure 7B). Anti-Ago2 RIP and double luciferase report experiments revealed that KRT17 was the direct target of miR-485-5p (*P* < 0.05) (Figure 7C-E). The Q-PCR results proved that the level of miR-485-5p was increased after miR-485-5p mimic treatment (*P* < 0.05) (Figure 7F). Similarly, it was discovered that KRT17 expression was descend after miR-485-5p mimic treatment, while KRT17 expression was rise after overexpression of KRT17 treatment in the miR-485-5p mimic treatment cell (*P* < 0.05) (Figure 7G).

### Influence of miRNA-485-5p regulating KRT17 on apoptosis and invasion of Pancreatic Cancer (PC) cells

**Figure 8:**
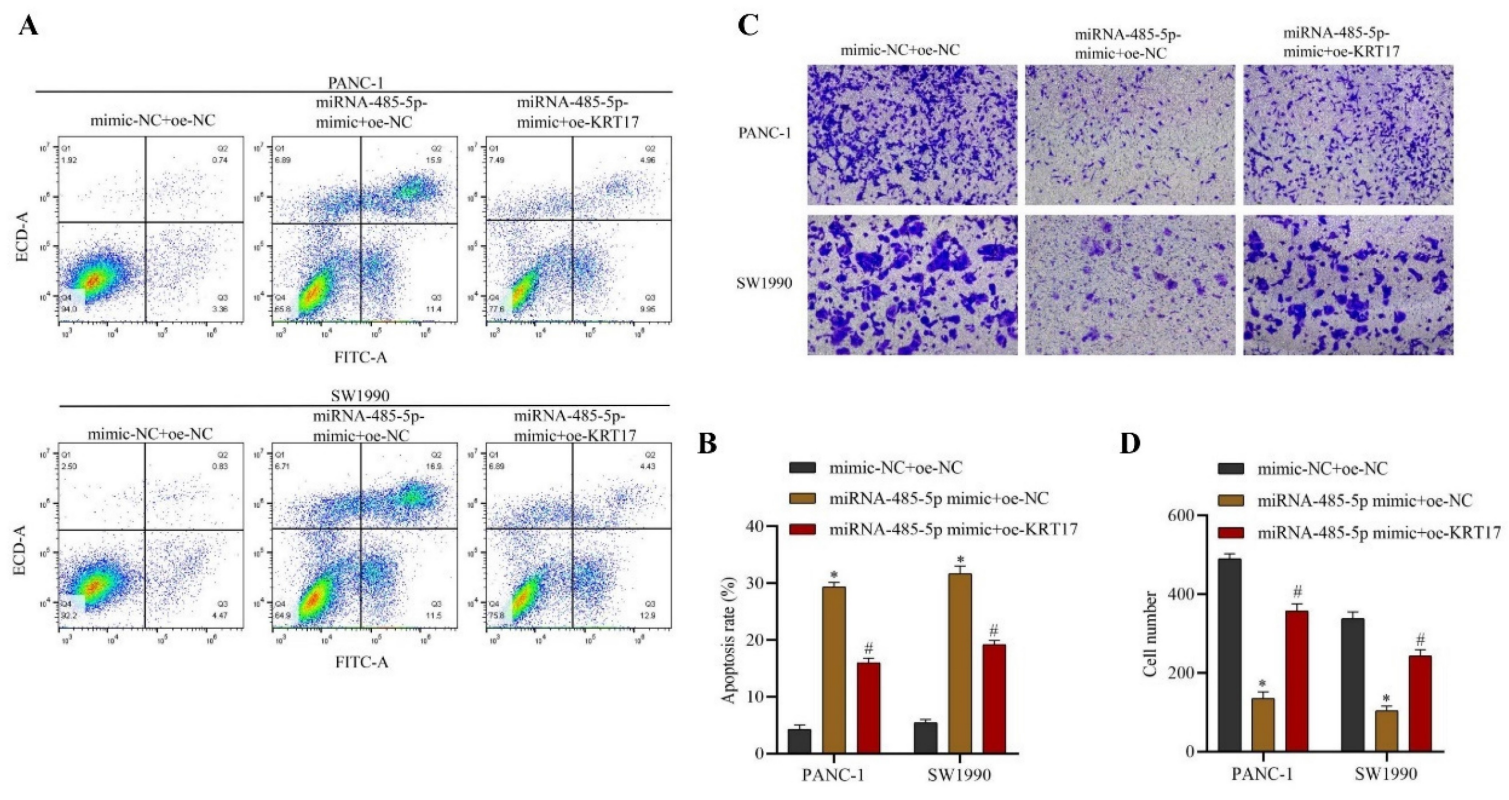
Influence of KRT17 regulation by miRNA-485-5p on the level of apoptosis and invasion of cells. **A** The regulatory apoptosis effect of miRNA-485-5p on KRT17 expression was studied by flow cytometry. **B** The statistical plot of apoptosis rates of cells. **C** Effect of KRT17 regulation by miRNA-485-5p on cell invasion. **D** The statistical plot of the invasion number of cells. ^*^P < 0.05 vs. mimic-NC+oe-NC. ^#^*P* < 0.05 vs. miRNA-485-5p mimic+oe-NC.

The flow cytometry and Transwell experiment result confirmed that the apoptosis level of the miRNA-485-5p mimic+oe-NC group was increased than NC group, whilst the invasive ability of the miRNA-485-5p mimic+oe-NC group was significantly reduced than NC group. Upregulation of the expression of KRT17 effectively weaken the impact of miRNA-485-5p on apoptosis and invasive ability, that is, the apoptosis level of the miRNA-485-5p mimic+oe-KRT17 group was much lower than that of the miRNA-485-5p mimic+oe-NC group, the invasive ability was dramatically increased (P < 0.05) (Figure 8A-D).

### Effects of miRNA-485-5p regulating KRT17 on proliferation and colony formation of pancreatic cancer (PC) cells

**Figure 9:**
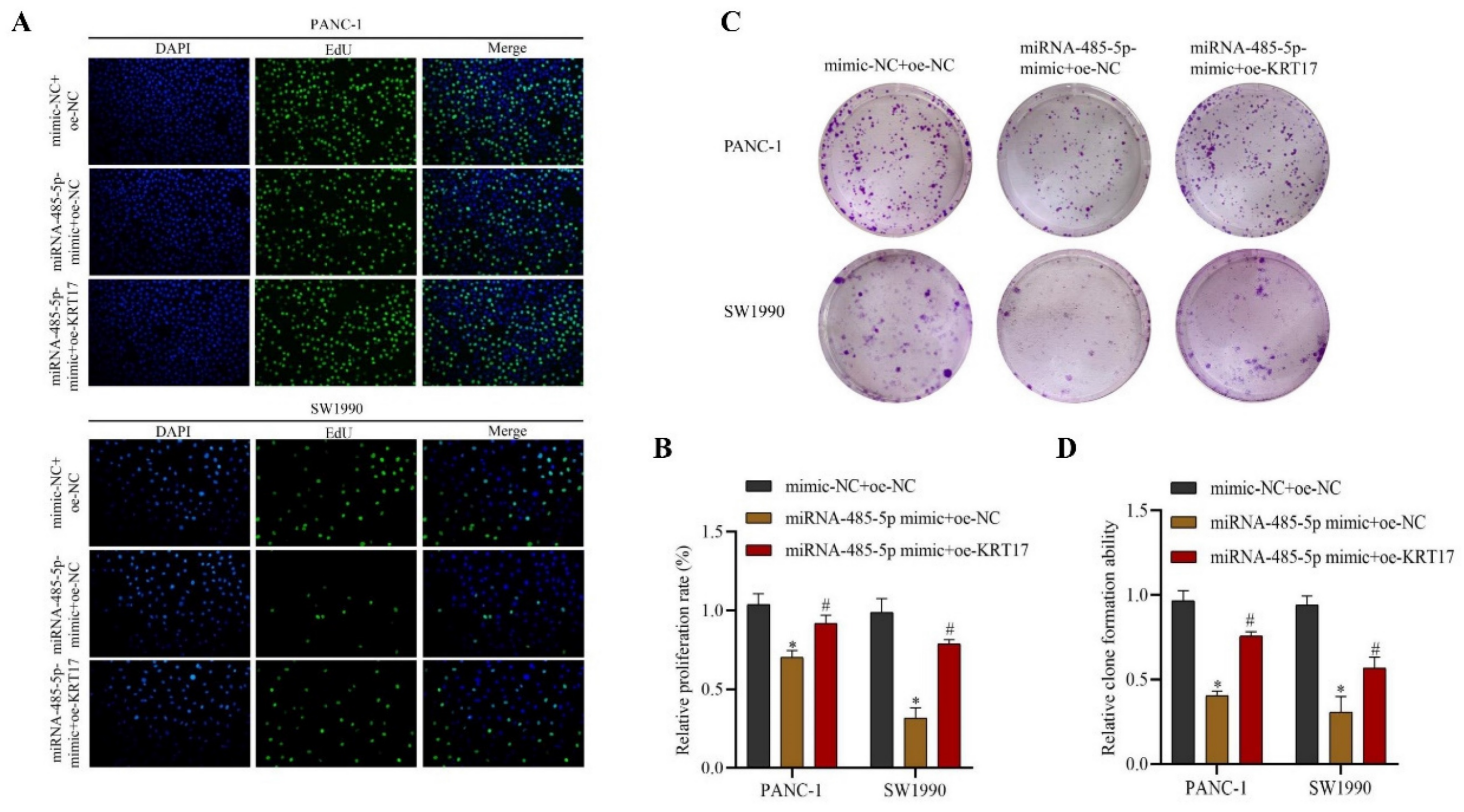
Regulation of KRT17 by miRNA-485-5p impairs cell proliferation and clonogenic ability in cells. **A** EdU experiments were performed to test the effect of KRT17 regulation by miRNA-485-5p on the proliferative capacity of cells. **B** The statistics plot of cell-associated proliferation rates for cells. **C** The cloning experiments were performed to test the effect of krt17 regulation by miRNA-485-5p on the clonogenic ability of cells. **D** The statistical chart of the clone formation ability of cells. ^*^P < 0.05 vs. mimic-NC+oe-NC. ^#^*P* < 0.05 vs. miRNA-485-5p mimic+oe-NC.

EDU and cloning tests disclosed that the growth and clonality of miRNA-485-5p mimic+oe-NC group in PANC-1 and SW1990 were lower than those in the mimic-NC+oe-NC group (*P* < 0.05). The miRNA-485-5p mimic+oe-KRT17 group substantially boosted cell proliferation and clone formation ability compared to the miRNA-485-5p mimic+oe-NC group (P < 0.05) (Figure 9A-D).

## Discussion

TCGA database analysis indicated that the expression of KRT17 was up-regulated in PC tissues, and the overexpression of KRT17 decreased the survival rate of PC patients The TargetScan database accompanied by dual luciferase and RIP experiments verified the targeting of KRT17 by miRNA-485-5p. The results of Q-PCR detection showed that KRT17 was strongly expressed while miRNA-485-5p was low in PC samples. Silencing KRT17 or overexpression of miRNA-485-5p in cells significantly suppress invasion, clone formation, and proliferation, and promoted apoptosis, whereas upregulation of KRT17 expression reverses miRNA-485-5p function. Simultaneously, results from western blot detection demonstrated that silencing KRT17 had an impact on the expression of cyclinD1, CDK1, and CDK2 proteins in PC cells as well as the FAK/Src/ERK signal pathway. These results imply that PC occurrence and development are significantly influenced by the regulation of KRT17 by miRNA-485-5p.

KRT17 is predominantly expressed in the human epidermis and its appendages. Prior research has linked unusual KRT17 expression to cell proliferation and tumorigenesis 29, 30. For instance, the expression levels were up-regulated of KRT17 encouraging cell proliferation and tumorigenesis in skin tumors, oral and cervical squamous cell carcinoma, gastric and lung cancer, and other tumors 31-35. Therefore, this study discovered that poor prognosis for PC patients with high content of KRT17. The colony formation, proliferation, and invasive ability of cells were attenuated after down-regulating the content of KRT17 in SK-MES-1 (lung cancer cell line) 36.

In this study, KRT17 expression in pancreatic cancer cell lines (PANC-1 and SW1990) was up-regulated about normal pancreatic cells (H6c7). Silencing KRT17 in cells dramatically decreased cell invasion, proliferation, and cloning, and enhanced cell apoptosis. As we all know, a tumor is a type of cell cycle disorder and the efficacy of various genes will affect the mechanism of the cell cycle to form a tumor 37, 38. Several studies have determined that silencing KRT17 caused cell cycle arrest in G0/G1 phase, suggesting that KRT17 might affect the cycle regulation of tumor cells [39). Cell cycle-dependent protein kinases (CDKs) belong to protein kinases 40. CDK regulates the cell cycle by applying to Ser/Thr protein and collaborates with cyclin (Cyclin), the engine of the cell cycle 41. CDK2 binds with CyclinA to promote cells into the G2 phase during the cell cycle 42,43. CDK1 binds with CyclinB, causing cells to advance from G2 to the M phase and complete cell division 42, 43. In this study, the GEPIA database indicated that KRT17 was positively correlated with cyclinD1 (CCND1) (*P*=1.4e-09), CDK1 (*P*=0.0057), and CDK2 (*P*=0.035) in PC. The results of western blot experiments indicated that silencing KRT17 in PC cell lines downregulated the content of CyclinD1, CDK1, and CDK2 proteins.

Focal adhesion kinase (FAK) plays a key role in intercellular signal transduction 44. Someone found that the expression and phosphorylation of FAK were tightly correlated with cancer metastasis and invasion 45. FAK can function as the upstream factor for Src tyrosine kinase 46. The phosphorylation of FAK in Y397 can form a binding site for the SH2 domain of Src (FAK/Src complex) 47. FAK/Src has been demonstrated in studies to regulate the biological function of many kinds of cells through the ERK pathway 48. Western blot detection revealed that silent KRT17 sharply down-regulated the expression of p-FAK, p-Src, and p-ERK protein in PANC-1 and SW1990 cells in this study (*P* < 0.05). The outcome revealed that KRT17 altered the biological functions of PC cells by inhibiting the FAK/Src/ERK signal pathway.

Studies have shown that miRNA can influence many kinds of malignant biological processes in PC cells 49. As a result, the search for miRNA related to the occurrence and progression of PC is critical to reveal its pathogenesis and improve the efficacy of targeted therapy 50. MiRNA-485-5p targeted KRT17 was found by bioinformatics analysis along with double luciferase and RIP experiments in this study. Additionally, Q-PCR results identified low levels of miR-485-5p in PC tumor tissues (n = 20). Upregulation of the expression of miRNA-485-5p in PANC-1 versus SW1990 cells downregulated KRT17 expression considerably. Studies have shown that levels of miRNA-485-5p are lower in colorectal cancer cells, and upregulating its expression can inhibit cancer cell invasion and proliferation. As per the findings of this research, upregulation of miR-485-5p restrain KRT17 overexpression in pancreatic cancer cells line, inhibiting the biological functions of cell invasion, proliferation, and cloning and promoting cell apoptosis. Overexpression of KRT17 in response to miRNA-485-5p overexpression effectively reversed the regulatory effect of miRNA-485-4p on cell biological function. MiRNA-485-5p is recognized to take part in PC by regulating KRT17.

## Conclusion

Our current research indicates a vital signal cascade of miR-485-5p/KRT17/FAK/Src/ERK, which affects the biological function of PC cells. Moreover, it provides fresh perspectives for revealing the pathogenesis of PC and may act as an underlying target for clinical targeted therapy of PC.

## Figures and Tables

**Table 1:** Primer sequences

Gene	Primer sequence (5ʹ-3ʹ)
KRT17	F: CAACACTGAGCTGGAGGTGA
	R: AACTTGGTGCGGAAGTCATC
miRNA-485-5p	F: TCAGAGGCTGGCCGTGAT
	R: GTGCAGGGTCCGAGGTAT
GAPDH	F: TGACTTCAACAGCGACACCCA
	R: CACCCTGTTGCTGTAGCCAAA
U6	F: TGCGGGTGCTCGCTTCGGCAGC
	R: CAGT GCAGGGTCCGAGGTAT
